# Complete genome sequence of *Actinobacillus equuli* subspecies *equuli* ATCC 19392^T^

**DOI:** 10.1186/s40793-015-0009-x

**Published:** 2015-06-07

**Authors:** Barbara F Huang, Andrew M Kropinski, Adina R Bujold, Janet I MacInnes

**Affiliations:** 1 Department of Pathobiology, University of Guelph, Ontario Veterinary College, Ontario N1G 2 W1, Canada

**Keywords:** Actinobacillus equuli subsp. equuli, Sleepy foal disease, Joint ill disease, Commensal, Equine

## Abstract

*Actinobacillus equuli* subsp. *equuli* is a member of the family *Pasteurellaceae* that is a common resident of the oral cavity and alimentary tract of healthy horses. At the same time, it can also cause a fatal septicemia in foals, commonly known as sleepy foal disease or joint ill disease. In addition, *A. equuli* subsp. *equuli* has recently been reported to act as a primary pathogen in breeding sows and piglets. To better understand how *A. equuli* subsp. *equuli* can cause disease, the genome of the type strain of *A. equuli* subsp. *equuli,* ATCC 19392^T^, was sequenced using the PacBio RSII sequencing system. Its genome is comprised of 2,431,533 bp and is predicted to encode 2,264 proteins and 82 RNAs.

## Introduction

*Actinobacillus equuli**subsp*. *equuli*, previously known as ‘*Bacillus**viscosum-equi*’, or ‘*Shigella**equirulis*’, is a common resident of the oral flora of healthy horses, as well as that of the alimentary and genital tracts [1],[2]. It has also been reported to be present in other host species such as mice, seemingly without ill effect [3] and on rare occasions, has been transmitted through bite wounds to humans [4]. *A. equuli**subsp*. *equuli* is the etiological agent of sleepy foal disease, an acute form of fatal septicemia in neonatal foals that may progress to a chronic form, joint ill disease, producing lesions in the kidneys, joints, and lungs [5]-[8]. Horses with *A. equuli* infection can present with arthritis, bronchitis, pneumonia, pleuritis, peritonitis, sepsis, endocarditis, pericarditis, nephritis, meningitis, metritis, and abortion [7],[9]-[12]. *A. equuli**subsp*. *equuli* was previously proposed to act as a secondary pathogen in foals; however, a recent study by Layman and colleagues [13] has revealed that *A. equuli**subsp*. *equuli* has the potential to act as a primary pathogen given favourable conditions. Recently, it has been reported to also be a primary pathogen in sows and piglets [14],[15].

The hemolytic counterpart of this bacterium, *A. equuli**subsp*. *haemolyticus**,* is isolated more frequently from the respiratory tract rather than the oral cavity. It can also cause septicemia and sequelae such as arthritis and meningitis, respiratory tract infections, and mare reproductive loss syndrome [8],[10],[16].

The similar colonial morphology and biochemical markers and shared 16S rRNA sequences make differentiation of *A. equuli* from *Actinobacillus suis* difficult [8]. In addition, little is known about the virulence factors of *A. equuli**subsp*. *equuli**.* To be better able to identify and to improve our understanding of the mechanism of pathogen-host interactions [7], the genome of the type strain *A. equuli**subsp*. *equuli* strain http://ATCC 19392http://T was sequenced. This strain was isolated from foal blood and deposited in the American Type Culture Collection by the Equine Research Station (New Market, UK) in 1953 [17].

## Organism information

### Classification and features

**Figure 1:**
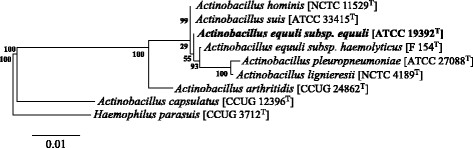
Phylogenetic tree based on 16S rRNA sequences of *Actinobacillus sensu stricto* species plus *A. capsulatus* and *H. parasuis* as outgroups. *A. equuli* subsp. *equuli* is indicated in bold*.* The RDP aligner, which applies the Jukes-Cantor corrected distance model to align sequences, and the RDP Tree Builder, which implements the Weighbor algorithm [36] for tree construction were used. Tree building also involved a bootstrapping process in which the values to the left of the branches illustrate the frequency of occurrence of a branch in 100 replicates [37].

As a member of the genus *Actinobacillus**,**A. equuli**subsp**.**equuli* belongs to the family *Pasteurellaceae*, class *Gammaproteobacteria*[18] (Table 1). Phylogenetic analysis using 16S rRNA sequences suggests that *A. equuli**subsp**.**equuli* is most closely related to *A. suis* and *A. hominis* (Figure 1).

**Table 1:** **Classification and features of** ***A. equuli subsp. equuli*** **ATCC 19392**^**T**^

**MIGS ID**	**Property**	**Term**	**Evidence code**^**a**^
	Classification	Domain *Bacteria*	TAS [21]
		Phylum *Proteobacteria*	TAS [22]
		Class *Gammaproteobacteria*	TAS [23],[24]
		Order *Pasteurellales*	TAS [25]
		Family *Pasteurellaceae*	TAS [26],[27]
		Genus *Actinobacillus*	TAS [28],[29]
		Species *Actinobacillus equuli*	TAS [28],[30],[31]
		Subspecies *Actinobacillus equuli* subsp. *equuli*	TAS [20]
		Type strain ATCC 19392^T^	
	Gram stain	Negative	TAS [32]
	Cell shape	Rods (pleomorphic)	TAS [33]
	Motility	Non-motile	TAS [33]
	Sporulation	Non-sporulating	TAS [33]
	Temperature range	Mesophile (20 - 44°C)	TAS [33]
	Optimum temperature	37°C	TAS [20]
	pH range	6.0 – 8.4	TAS [1]
	Carbon source	Saccharolytic	TAS [19]
MIGS-6	Habitat	Host, equine or swine upper respiratory tract, alimentary tract, and genital tract	TAS [4],[5],[19]
MIGS-6.3	Salinity	0.5% NaCl	NAS
MIGS-22	Oxygen requirement	Facultative anaerobe	TAS [19],[33]
MIGS-15	Biotic relationship	Commensal or opportunistic	TAS [14],[15]
MIGS-14	Pathogenicity	Variable	TAS [13]
MIGS-4	Geographic location	New Market, UK	TAS [17]
MIGS-5	Sample collection	1953	TAS [17]
MIGS-4.1 MIGS-4.2	Latitude	Not reported	
	Longitude	Not reported	
MIGS-4.4	Altitude	Not reported	

^a^Evidence codes - TAS: Traceable Author Statement; NAS: Non-traceable Author Statement (i.e., not directly observed for the living, isolated sample, but based on a generally accepted property for the species, or anecdotal evidence). These evidence codes are from the Gene Ontology project [34].

**Figure 2:**
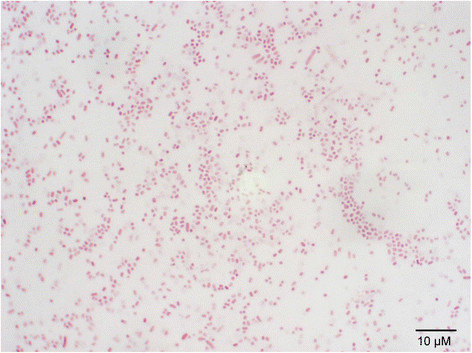
Gram stain of *A. equuli* subsp. *equuli* ATCC 19392^T^ at 1000 X magnification.

**Figure 3:**
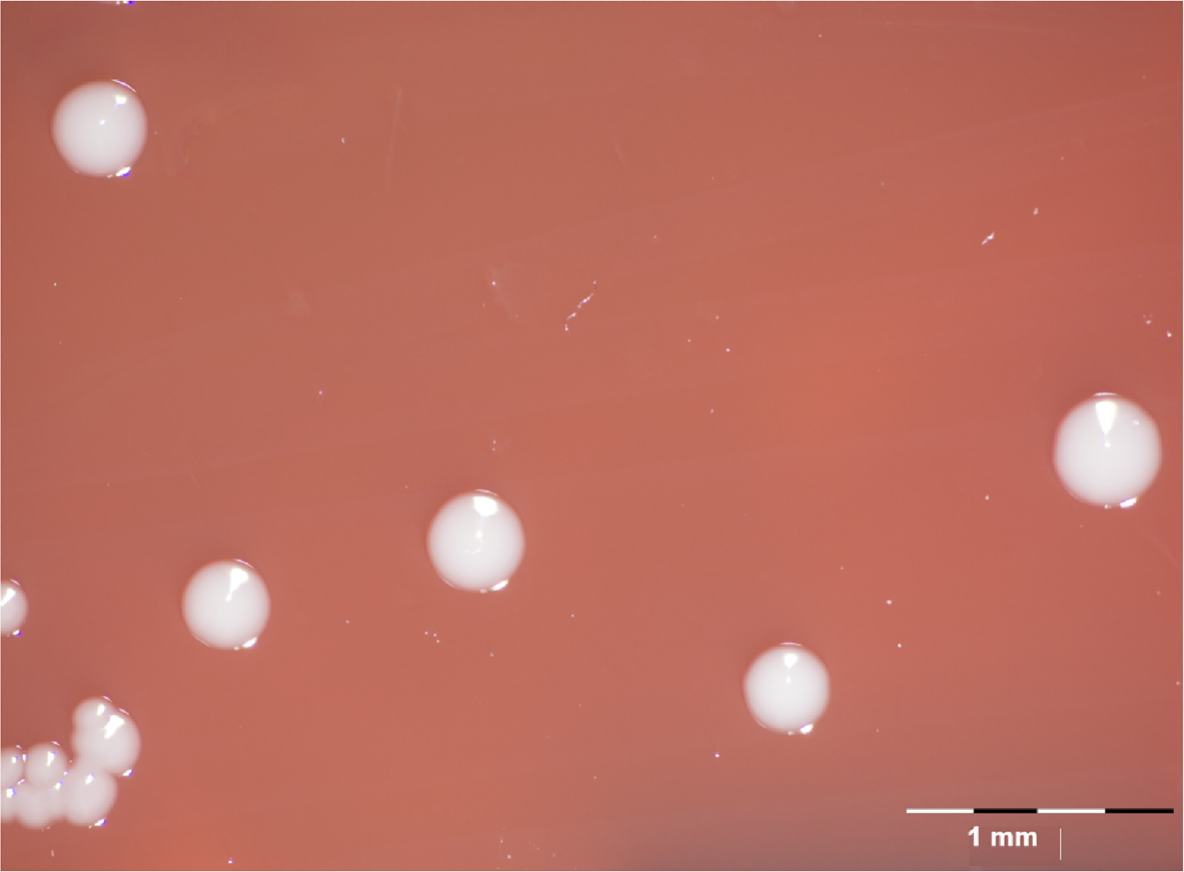
*A. equuli* subsp*. equuli* ATCC 19392^T^ colonies on sheep blood agar.

*A. equuli**subsp*. *equuli* is a small, Gram-negative, nonmotile, pleomorphic bacterium [15],[16],[19] (Figure 2). It is NAD-independent, nonhemolytic, and CAMP negative [15],[20]. *A. equuli**subsp*. *equuli* produces large amounts of extracellular slime that imparts sticky properties in solid and liquid media cultures [19],[31]*.* On nutrient or blood agar, smooth, grayish-white, circular colonies are produced with an average diameter of 1-2 mm after growth for 24 h [35] (Figure 3). On initial culture from clinical material, colonies are viscous and usually rough but become smooth in successive subcultures [1],[19]. Growth using liquid culturing methods has been reported to increase viability in comparison to solid media cultures, and viscosity is retained upon repeated subculturing [1],[19]. The usual temperature range for growth of this bacterium is 20-39°C, with an optimum at 37°C, though some *A. equuli**subsp*. *equuli* strains have been shown to grow at temperatures as high as 44°C [33]. Acid but not gas is produced from sucrose, mannitol, galactose, lactose, maltose, mannose, melibiose, trehalose, raffinose, and glycerol fermentation [19],[20],[33]. *A. equuli**subsp*. *equuli* is capable of reducing nitrate and produces α-galactosidase, α-glucosidase, β-xylosidase, urease, and oxidase [19],[20],[33].

## Genome sequencing information

### Genome project history

*A. equuli**subsp*. *equuli* was selected for sequencing because of its importance to the horse industry as the etiologic agent of sleepy foal disease and joint ill disease [7]. Sequencing was done at the McGill University and Génome Québec Innovation Centre (Montréal, QC, Canada) using the PacBio RS II DNA Sequencing System, and assembled using PacBio RS II software and Celera Assembler. *A. equuli**subsp*. *equuli* was annotated using the NCBI Prokaryotic Genome Annotation Pipeline. A summary of the project information and the Minimum Information about a Genomic Sequence is shown in Table 2[38].

**Table 2:** **Project information and its association with MIGS version 2.0 compliance**[38]

**MIGS ID**	**Property**	**Term**
MIGS-31	Finishing quality	Complete
MIGS-28	Libraries used	SMRTbell library
MIGS-29	Sequencing platforms	PacBio RS II
MIGS-31.2	Fold coverage	196x
MIGS-30	Assemblers	PacBio RS II, Celera
MIGS-32	Gene calling method	GeneMarkS+
	Locus Tag	ACEE
	Genbank ID	CP007715
	GenBank Date of Release	December 15, 2014
	GOLD ID	Gp0095186
	BIOPROJECT	PRJNA247050
MIGS-13	Source Material Identifier	ATCC 19392^T^
	Project relevance	Equine and swine pathogenesis

### Growth conditions and genomic DNA preparation

*A. equuli**subsp*. *equuli* was grown from a frozen (-70°C) seed stock on sheep blood agar plates overnight in an atmosphere of 5% CO_2_ at 37°C. After subculture, well-isolated colonies were used for genomic DNA isolation. Cells were lysed using modified B1 (150 mM Tris · Cl, 50 mM EDTA, 0.5% Tween®-20, 0.5% Triton X-100, pH 8.0) and B2 (750 mM NaCl, 50 mM MOPS, 15% isopropanol, 0.15% Triton X-100, pH 7.0) buffers. DNA was then column purified using a QIAGEN Plasmid Midi Kit (Qiagen, Germany) following manufacturer's protocol for binding and elution. The resultant DNA preparation was characterized using a NanoDrop model ND1000 Spectrophotometer and was diluted to a concentration of ~0.47 mg/μl.

### Genome sequencing and assembly

Single Molecule, Real-Time DNA sequencing (Pacific Biosciences) [39] was done to obtain the genome sequence of the *A. equuli**subsp*. *equuli*http://ATCC 19392http://T. A total of 133,616 raw subreads were generated with an average length of 4,348 bp using two SMRT Cells in a PacBio RSII sequencer. The resultant subread length cutoff value, 29.42, was used in the Basic Local Alignment with Successive Refinement step [40] where short reads were used to correct for errors on long reads [39]. The corrected reads were assembled into contigs according to the Hierarchical Genome Assembly Process (HGAP) workflow using the Celera Assembler and refined using BLASR to align raw reads on contigs [39]. Final processing was conducted using Quiver, a variant calling algorithm, to generate high quality consensus sequences [39]. There were a total of 4,777 corrected reads with an average length of 7,804 bp and a final product of one contig.

### Genome annotation

Genes were identified using the NCBI Prokaryotic Genome Annotation Pipeline. The prediction software, GeneMark, is integrated into the pipeline and performs unsupervised gene finding using heuristic Markov Models [41]. Additional gene prediction analysis and functional annotation was performed within the Integrated Microbial Genomes (IMG) platform [42] developed by the Joint Genome Institute [43] (Table 3).

**Table 3:** Genome statistics

**Attribute**	**Value**	**% of total**^**a**^
Genome size (bp)	2,431,533	100.00
DNA coding (bp)	2,169,474	89.22
DNA G + C (bp)	979,048	40.26
DNA scaffolds	1	100.00
Total genes^b^	2,264	100.00
Protein coding genes	2,182	96.38
RNA genes	82	3.62
Pseudo genes^c^	11	0.49
Genes in internal clusters	1,466	64.75
Genes with function prediction	1,993	88.03
Genes assigned to COGs	1,752	77.39
Genes with Pfam domains	1,964	86.75
Genes with signal peptides	235	10.38
Genes with transmembrane helices	508	22.44
CRISPR repeats	2	0.08

^a^The total is based on either the size of the genome in base pairs or the total number of protein coding genes in the annotated genome.

^b^Also includes 11 pseudogenes and one other RNA gene that does not belong to rRNA or tRNA categories.

^c^Pseudogenes are not additive under total genes and may be counted as either protein coding or RNA genes.

### Genome properties

The genome of *A. equuli**subsp*. *equuli* is a single circular chromosome that is 2,431,533 bp in length with a G + C content of approximately 40.3%. It is predicted to contain 2,264 genes, of which 2,182 code for proteins and 82 for RNA; 11 pseudogenes are also present (Table 3 and Figure 3). Approximately 3/4 of the predicted genes can be assigned to one of 25 functional COG categories (Table 4). Of particular note with regard to virulence are several lipopolysaccharide genes predicted to encode biosynthetic enzymes for the O-antigen and lipid A components. Adhesins of different types were observed including several autotransporters; a tight adherence locus; prepilins, and fimbriae; a filamentous hemagglutinin homolog was also detected. In addition, several putative iron acquisition systems are present including those for siderophores, hemoglobin and transferrin. A number of toxin and hemolysin genes were also identified including an *aqxCABD* operon, although compared to the *aqxCABD* of *A. equuli**subsp*. *haemolyticus* there are many point mutations and sizable deletions at both ends of the *aqxA* gene. Other regions of particular interest include an integron and Mu-like phage, identified using PHAST [44].

**Table 4:** Number of genes associated with general COG functional categories

**Code**	**Value**	**% age**	**Description**
J	159	8.49	Translation
A	1	0.05	RNA processing and modification
K	94	5.02	Transcription
L	105	5.61	Replication, recombination and repair
B	-	-	Chromatin structure and dynamics
D	24	1.28	Cell cycle control, mitosis and meiosis
Y	-	-	Nuclear structure
V	18	0.96	Defense mechanisms
T	35	1.87	Signal transduction mechanisms
M	136	7.26	Cell wall/membrane biogenesis
N	4	0.21	Cell motility
Z	-	-	Cytoskeleton
W	2	0.11	Extracellular structures
U	44	2.35	Intracellular trafficking and secretion
O	92	4.91	Posttranslational modification, protein turnover, chaperones
C	117	6.25	Energy production and conversion
G	126	6.73	Carbohydrate transport and metabolism
E	176	9.40	Amino acid transport and metabolism
F	63	3.36	Nucleotide transport and metabolism
H	108	5.77	Coenzyme transport and metabolism
I	45	2.40	Lipid transport and metabolism
P	133	7.10	Inorganic ion transport and metabolism
Q	14	0.75	Secondary metabolites biosynthesis, transport and catabolism
R	194	10.36	General function prediction only
S	183	9.77	Function unknown
-	512	22.61	Not in COGs

### Insights from the genome sequence

**Figure 4:**
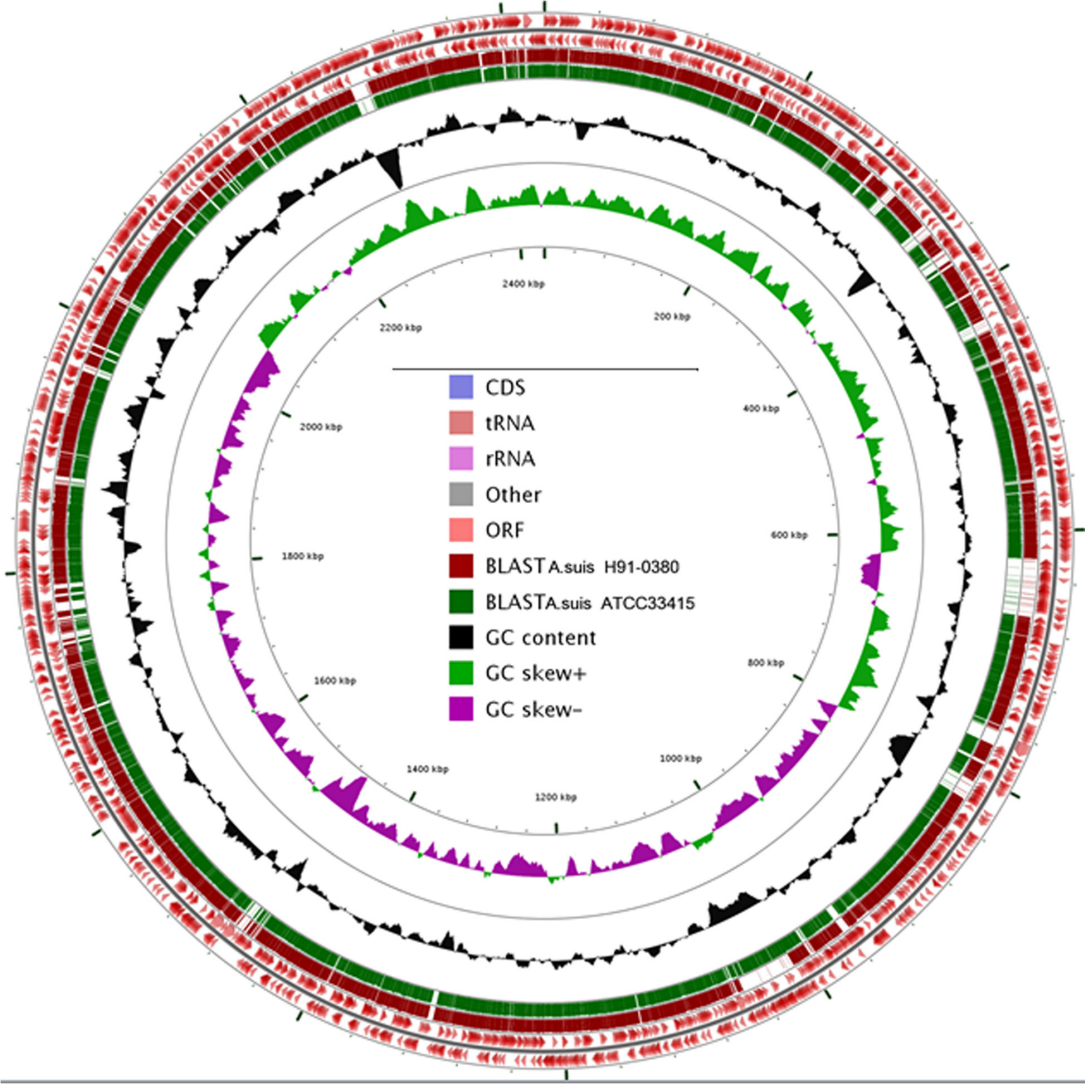
Circular map of the *A. equuli* subsp. *equuli* ATCC 19392^T^ genome generated using the CGView Server [49]. From the outside to the center: coding sequences (CDSs) in positive strand, reverse strand CDSs, BLASTN versus *A. suis* strain H91-0380 (CP003875), BLASTN versus *A. suis* ATCC 33415 (CP009159), GC content, and GC skew.

Given the marked similarities of *A. equuli* and *A. suis* there has been some debate as to whether these organisms should be a single species. In the current study we determined that the *A. equuli**subsp*. *equuli* 16S genes are 99% identical to those of both *A. suis* H91-0380 and the *A. suis**type* strain, http://ATCC 33415, consistent with membership in the same species. Further, as can be seen in the circular maps below, the genome of *A. equuli**subsp*. *equuli* is very similar to that of *A. suis* again suggesting that *A. equuli**subsp**.**equuli* and *A. suis* might be the same species (Figure 4). On the other hand, when genomes of *A. suis* H91-0380 and *A. suis*http://ATCC 33415 were compared with that of *A. equuli**subsp*. *equuli* using the ANI calculator [45], the ANI value of both comparisons was 93.82%, which is lower than 95%, the recommended cutoff value for delineating species [46].

In-silico DNA-DNA hybridization, done using a Genome Blast Distance Phylogeny approach to generate genome based distance measures for phylogenetic inferences, also demonstrated differences between *A. equuli* and *A. suis*. The Genome-to-Genome Distance Calculator [47] revealed a distance of 0.0685 between *A. suis* H91-0380 and *A. equuli**subsp*. *equuli**,* with a DDH estimate of 51.40% +/- 2.66. A DDH similarity below 70% is interpreted as two species being distinct; 79% is used to discriminate between subspecies [48]. The DDH estimate exceeding the 70% species threshold was determined from logistic regression to be 23.14%. In terms of subspecies relatedness, the probability of exceeding the 79% threshold was 4.82% between *A. equuli**subsp*. *equuli* and *A. suis* H91-0380. The distance calculated between *A. suis*http://ATCC 33415 and *A. equuli**subsp*. *equuli* and their DDH estimate was 0.0681 and 51.60% +/- 2.66, respectively. The probability that DDH exceeded 70% and 79% for *A. suis*http://ATCC 33415 and *A. equuli**subsp*. *equuli* were 23.66% and 4.94%, respectively.

Taken together, these analyses are consistent with the notion that *A. suis* and *A. equuli**subsp*. *equuli* are related but distinct species, and care is needed to correctly identify them.

## Conclusions

*A. equuli**subsp*. *equuli* can induce fatal septicemia in foals resulting in significant economic losses in the equine industry; as well, *A. equuli**subsp*. *equuli* has recently been reported to cause septicemia in swine of all ages. Our analysis of the *A. equuli**subsp*. *equuli* genome indicates that *A. suis* and *A. equuli**subsp*. *equuli* are closely related yet distinct species. At the present time little is known about how *A. equuli**subsp*. *equuli* causes disease or the factors that control species and tissue tropism. More research including biological experiments is required to better understand the pathogenesis of *A. equuli* and it is hoped this reported genome sequence of *A. equuli**subsp*. *equuli*http://ATCC 19392http://T will provide vital information for such studies. In addition, pathway analysis and genome studies may help improve our understanding of host-pathogen interactions of *A. equuli**subsp*. *equuli* and other *Actinobacillus* species and aid in the design of diagnostic tools and antimicrobial agents.

## Competing interests

The authors declare that they have no competing interests.

## Authors’ contributions

JIM and AMK contributed to the conception and design of this project. BFH and AMK were involved in the acquisition and initial analysis of the data; BFH, AMK, ARB and JIM were involved in the interpretation of the data. BFH prepared the first draft of the manuscript. All authors were involved in its critical revision and have given final approval of the version to be published and agree to be accountable for all aspects of the work.
